# 5-(4-Fluoro­phen­yl)-2-[2-(5-phenyl-1,3-oxazol-2-yl)phen­yl]-1,3-oxazole

**DOI:** 10.1107/S1600536810031235

**Published:** 2010-08-21

**Authors:** Rodion Ilyashenko, Michał Wera, Jerzy Błażejowski, Andrey Doroshenko

**Affiliations:** a Department of Chemistry, Kharkov V. N. Karazin National University, 4 Svobody Sqr., Kharkov 61077, Ukraine; b Faculty of Chemistry, University of Gdańsk, Sobieskiego 18, 80-952 Gdańsk, Poland

## Abstract

In the title compound, C_24_H_15_FN_2_O_2_, the dihedral angles between the central benzene ring and the oxazole rings are 10.7 (6) and 64.1 (5)°. The dihedral angles between the oxazole rings and their pendant rings are 2.0 (3) and 24.3 (2)°. The F atoms are disordered over two sites with occupancies of 0.627 (3) and 0.373 (3) in the phenyl­ene–oxazol­yl–phenyl and in oxazol­yl–phenyl fragments, respectively. In the crystal structure, mol­ecules are linked through a network of C—H⋯F and weak π–π stacking inter­actions.

## Related literature

For background to the practical applications of 1,2-bis-(5-phenyl-oxazol-2-yl)benzene (*ortho*-POPOP) analogs (spectroscopic and fluorescence kinetics data), see: Doroshenko *et al.* (1996 ▶, 1999 ▶, 2000*a* ▶,*c* ▶, 2002*a* ▶), Kirichenko *et al.* (1998 ▶). For related structures, see: Doroshenko *et al.* (1994 ▶, 1997 ▶, 2000*b* ▶, 2002*b* ▶).
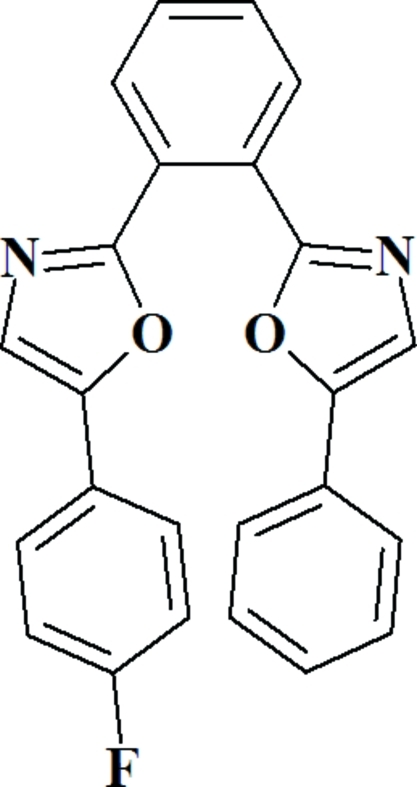


## Experimental

### 

#### Crystal data

C_24_H_15_FN_2_O_2_
*M* *_r_* = 382.38
Monoclinic, 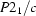

*a* = 9.3158 (3) Å
*b* = 10.8176 (3) Å
*c* = 18.9449 (5) Å
β = 100.571 (3)°
*V* = 1876.76 (9) Å^3^
*Z* = 4
Mo *K*α radiation
μ = 0.09 mm^−1^
*T* = 295 K
0.6 × 0.3 × 0.05 mm

#### Data collection

Oxford Diffraction Gemini R Ultra Ruby CCD diffractometer
Absorption correction: multi-scan (*CrysAlis RED*; Oxford Diffraction, 2008 ▶) *T*_min_ = 0.956, *T*_max_ = 0.991
16242 measured reflections
4263 independent reflections
2707 reflections with *I* > 2σ(*I*)
*R*_int_ = 0.024

#### Refinement

*R*[*F* ^2^ > 2σ(*F* ^2^)] = 0.048
*wR*(*F* ^2^) = 0.120
*S* = 1.04
4263 reflections
266 parameters
1 restraint
H-atom parameters constrained
Δρ_max_ = 0.11 e Å^−3^
Δρ_min_ = −0.17 e Å^−3^

### 

Data collection: *CrysAlis CCD* (Oxford Diffraction, 2008 ▶); cell refinement: *CrysAlis RED* (Oxford Diffraction, 2008 ▶); data reduction: *CrysAlis RED*; program(s) used to solve structure: *SHELXS97* (Sheldrick, 2008 ▶); program(s) used to refine structure: *SHELXL97* (Sheldrick, 2008 ▶); molecular graphics: *ORTEP-3 for Windows* (Farrugia, 1997 ▶); software used to prepare material for publication: *SHELXL97* and *PLATON* (Spek, 2009 ▶).

## Supplementary Material

Crystal structure: contains datablocks I, global. DOI: 10.1107/S1600536810031235/fb2208sup1.cif

Structure factors: contains datablocks I. DOI: 10.1107/S1600536810031235/fb2208Isup2.hkl

Additional supplementary materials:  crystallographic information; 3D view; checkCIF report

## Figures and Tables

**Table 1:** Hydrogen-bond geometry (Å, °)

*D*—H⋯*A*	*D*—H	H⋯*A*	*D*⋯*A*	*D*—H⋯*A*
C16—H16*A*⋯F1^i^	0.93	2.31	3.235 (2)	170
C28—H28*A*⋯F1^ii^	0.93	2.45	3.155 (2)	133

Symmetry codes: (i) 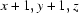; (ii) 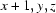.

**Table 2:** π–π inter­actions (Å, °) *Cg*1, *Cg*2 and *Cg*3 are the centroids of the C2–C7, O9/C8/C12/N11/C10 and C13–C18 rings, respectively. *CgI*⋯ *CgJ* is the distance between the ring centroids. The inter­planar angle is that between the planes of rings *I* and *J. CgI*_Perp is the perpendicuar distance of *CgI* from ring *J. CgI*_Offset is the distance between *CgI* and the perpendicular projection of *CgJ* on the ring *I*.

*I*	*J*	*CgI*⋯*CgJ*	Inter­planar angle	*CgI*_Perp	*CgI*_Offset
2	1^iii^	3.818 (1)	2.0 (1)	3.505 (1)	1.514
1	2^iii^	3.818 (1)	2.0 (1)	3.546 (1)	1.415
2	3^iv^	3.860 (1)	10.9 (1)	3.803 (1)	0.661
3	2^iv^	3.860 (1)	10.9 (1)	3.762 (1)	0.864

Symmetry codes: (iii) 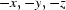; (iv) 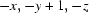.

**Fig. 1.**
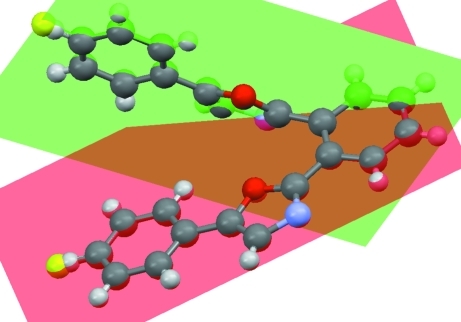
Two quasi-planar fragments in the molecule of the title compound in the crystalline state: phenylene-oxazolyl-phenyl mean plane is shown in red, while as oxazolyl-phenyl one - in green (the interplanar angle is ~75°).

**Fig. 2.**
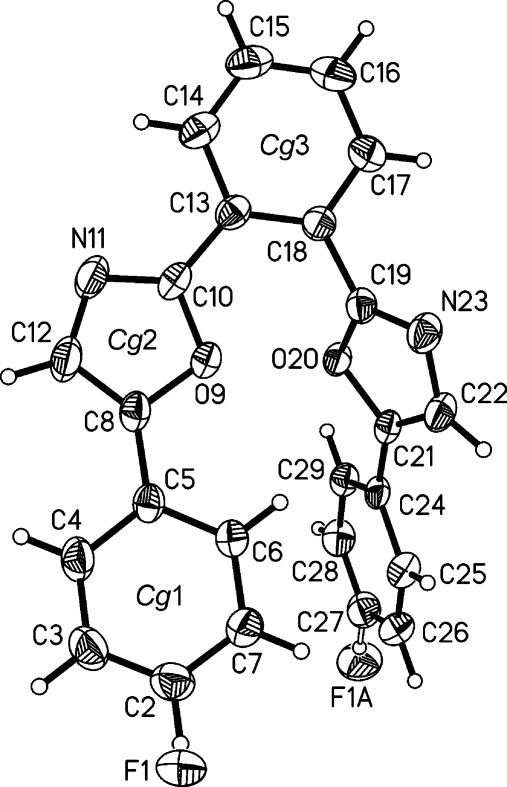
The title molecule with the atom numbering scheme. The displacement ellipsoids are drawn at the 25% probability level. Cg1, Cg2 and Cg3 denote the ring centroids.

**Fig. 3.**
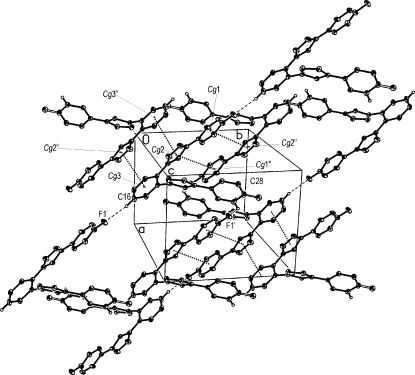
The arrangement of the molecules in the crystal structure, viewed approximately along the c axis. The C–H···F interactions are represented by the dashed lines and the π-electron ring-π-electron ring interactions by the dotted lines. The H atoms not involved in interactions have been omitted [symmetry codes: (i) x + 1, y + 1, z; (ii) x + 1, y, z; (iii) -x, -y, -z; (iv) -x, -y + 1, -z.]

**Fig. 4.**
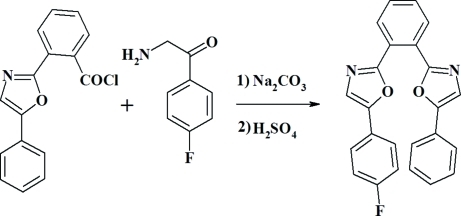
The scheme of the synthesis of the title compound. General procedure is analogous to that of Doroshenko (1994, 2000b, 2002b).

## supplementary crystallographic information

### Comment

Derivatives of 1,2-bis-(5-phenyl-oxazol-2-yl)benzene (*ortho*-POPOP) belong to the class of organic molecules, which exhibit efficient fluorescence with abnormally high Stokes shift (Doroshenko, 1996, 1999, 2000*b*). These molecules are prospective for their practical application as fluorescent probes, labels and chemosensors (Doroshenko, 2002*a*). Presence of two bulky heterocyclic substituents in 1,2-positions of the *ortho*-POPOP central benzene ring results in a significant sterical hindrance. This hindrance is manifested by a prominent non-planarity of *ortho*-POPOPs both in the crystalline state (Doroshenko, 1994) and in solutions (Doroshenko, 1996, 2002*a*). All the already examined crystal structures of the *ortho*-analogs of POPOP are characterized by essentially different angles between the planes of the central phenylene and each of the attached heterocyclic rings. Thus, two quasi-planar fragments containing two and three aromatic or heteroaromatic rings could be defined for the *ortho*-POPOPs molecules in crystals (Doroshenko, 1994, 1997, 2000*b*, 2002*b*).

The same arrangement is typical for the newly synthesized fluoro-substituted representative of this series (Fig. 1). The fluorine atom and the corresponding hydrogen are significantly disordered in the crystal structure over two sites with probabilities 0.627 (3) and 0.373 (3) and *vice versa* (Fig. 2). The title molecules are linked through a network of C–H···F hydrogen bonds (Tab. 1) and π-electron ring–π-electron ring interactions in the solid state (Fig. 3, Tab. 2).

### Experimental

Synthesis of the title compound was conducted according to the procedure presented on Fig. 4:

1 g (0.0038 mol) of 2-(5-phenyl-oxazol-2-yl)benzoic acid (Doroshenko, 1994, 2000*b*, 2002*b*) was boiled in 15 ml of thionyl chloride during 2 h. After adding of 20 ml of *ortho*-xylene the excess of thionyl chloride was distilled off. The resulting solution of 2-(5-phenyl-oxazol-2-yl)benzoic acid chloride in xylene was added to the solution of 0.72 g (0.0038 mol) of 4-*F*-ω-aminoacetophenone hydrochloride in 20 ml of water. The reaction mixture was gradually basified to pH~9 by the saturated aqueous sodium carbonate while intensive stirring for 1 h. The resulting solid was filtered, washed by distilled water, dried, dissolved in 25 ml of concentrated sulfuric acid and left on for 7 h at room temperature. Then the reaction mixture was poured on ice and filtered to give 0.92 g (0.0024 mol, 60%) of the final product (1-(5-[4'-*F*-phenyl]-oxazol-2-yl)-2-(5-phenyloxazol-2-yl)-benzene) as colorless solid (m.p. 98–99°C). The crystals were obtained by crystallization from hexane.

The necessary precursors have been prepared from the commercially available chemicals by the following procedures:

4-*F*-ω-Br-Acetophenone: 16 g (0.1 mol) of bromine was added dropwise to the solution 13.8 g (0.1 mol) of 4-*F*-acetophenone in 50 ml of ethanol at 30–40°C while stirring. After decolorization of the reaction mixture, ethanol was removed *in vacuo*. The resulting light yellow oily liquid was washed several times with distilled water to remove the traces of hydrobromic acid and used in the following synthesis without additional purification. Yield 19.1 g (0.088 mol, 88%).

4-*F*-ω-Amino-acetophenone hydrochloride: 6.5 g (0.046 mol) of hexamethylenetetramine was added portionwise at continuous stirring to the solution of 10 g (0.046 mol) of 4-*F*-ω-Br-acetophenone in 70 ml of chloroform. The reaction mixture was stirred during 5 h, then the precipitated solid was filtered, washed with chloroform, dried and mixed with 25 ml of concentrated HCl in 70 ml of ethanol. The mixture was stirred until the complete dissolution was reached and then it was kept for 7 h at room temperature. The precipitated ammonium chloride was filtered off, the filtrate was concentrated *in vacuo* and the resulted solid was boiled with 50 ml of acetone during 1 h, cooled to the room temperature and filtered off to give 7.2 g (0.038 mol, 83%) of colorless powder of 4-*F*-ω-aminoacetophenone hydrochloride (m.p. 169–171°C).

### Refinement

All the H atoms could be seen in the difference density maps with the exception of the atoms H2X and H27X that are disordered with F1 and F1A, respectively. However, the H atoms have been situated into the idealized positions with C—H = 0.93 Å and constrained to ride on their parent atoms with *U*_iso_(H) = 1.2*U*_eq_(C). The distances C2-F1 and C27-F1A have been restrained by *SADI* command of *SHELXL97* (Sheldrick, 2008). The value of an effective standard deviation of *SADI* was 0.001. The sum of the occupations of F1 and F1A was constrained to equal to 1 as it follows from the reaction (Fig. 3). Both fluorines were refined anisotropically.

### Crystal data

**Table:**

C_24_H_15_FN_2_O_2_	*F*(000) = 792
*M**_r_* = 382.38	*D*_x_ = 1.353 Mg m^−^^3^
Monoclinic, *P*2_1_/*c*	Melting point = 371–372 K
Hall symbol: -P 2ybc	Mo *K*α radiation, λ = 0.71073 Å
*a* = 9.3158 (3) Å	Cell parameters from 3303 reflections
*b* = 10.8176 (3) Å	θ = 3.3–27.5°
*c* = 18.9449 (5) Å	µ = 0.09 mm^−^^1^
β = 100.571 (3)°	*T* = 295 K
*V* = 1876.76 (9) Å^3^	Plate, colorless
*Z* = 4	0.6 × 0.3 × 0.05 mm

### Data collection

**Table:**

Oxford Diffraction GEMINI R ULTRA Ruby CCD diffractometer	4263 independent reflections
Radiation source: Enhance (Mo) X-ray Source	2707 reflections with *I* > 2σ(*I*)
graphite	*R*_int_ = 0.024
Detector resolution: 10.4002 pixels mm^-1^	θ_max_ = 27.5°, θ_min_ = 3.3°
ω–scan	*h* = −12→8
Absorption correction: multi-scan (*CrysAlis RED*; Oxford Diffraction, 2008)	*k* = −14→13
*T*_min_ = 0.956, *T*_max_ = 0.991	*l* = −24→23
16242 measured reflections	

### Refinement

**Table:**

Refinement on *F*^2^	Primary atom site location: structure-invariant direct methods
Least-squares matrix: full	Secondary atom site location: difference Fourier map
*R*[*F*^2^ > 2σ(*F*^2^)] = 0.048	Hydrogen site location: difference Fourier map
*wR*(*F*^2^) = 0.120	H-atom parameters constrained
*S* = 1.04	*w* = 1/[σ^2^(*F*_o_^2^) + (0.057*P*)^2^ + 0.132*P*] where *P* = (*F*_o_^2^ + 2*F*_c_^2^)/3
4263 reflections	(Δ/σ)_max_ < 0.001
266 parameters	Δρ_max_ = 0.11 e Å^−^^3^
1 restraint	Δρ_min_ = −0.17 e Å^−^^3^

### Special details

**Table:**

Geometry. All esds (except the esd in the dihedral angle between two l.s. planes) are estimated using the full covariance matrix. The cell esds are taken into account individually in the estimation of esds in distances, angles and torsion angles; correlations between esds in cell parameters are only used when they are defined by crystal symmetry. An approximate (isotropic) treatment of cell esds is used for estimating esds involving l.s. planes.
Refinement. Refinement of *F*^2^ against ALL reflections. The weighted *R*-factor *wR* and goodness of fit *S* are based on *F*^2^, conventional *R*-factors *R* are based on *F*, with *F* set to zero for negative *F*^2^. The threshold expression of *F*^2^ > σ(*F*^2^) is used only for calculating *R*-factors(gt) *etc*. and is not relevant to the choice of reflections for refinement. *R*-factors based on *F*^2^ are statistically about twice as large as those based on *F*, and *R*- factors based on ALL data will be even larger.

### Fractional atomic coordinates and isotropic or equivalent isotropic displacement parameters (Å^2^)

**Table:**

	*x*	*y*	*z*	*U*_iso_*/*U*_eq_	Occ. (<1)
C2	−0.2973 (2)	−0.04508 (16)	0.07969 (11)	0.0836 (5)	
H2X	−0.3553	−0.1002	0.0996	0.100*	0.373 (3)
C3	−0.3040 (2)	−0.04438 (19)	0.00717 (11)	0.0897 (6)	
H3A	−0.3658	−0.0985	−0.0221	0.108*	
C4	−0.21904 (19)	0.03660 (18)	−0.02205 (9)	0.0782 (5)	
H4A	−0.2227	0.0367	−0.0714	0.094*	
C5	−0.12753 (16)	0.11865 (14)	0.02066 (8)	0.0567 (4)	
C6	−0.12191 (19)	0.11481 (14)	0.09413 (8)	0.0637 (4)	
H6A	−0.0595	0.1680	0.1238	0.076*	
C7	−0.2071 (2)	0.03362 (15)	0.12375 (10)	0.0765 (5)	
H7A	−0.2035	0.0321	0.1731	0.092*	
C8	−0.04037 (17)	0.20440 (15)	−0.01170 (8)	0.0610 (4)	
O9	0.05304 (11)	0.28136 (9)	0.03280 (5)	0.0564 (3)	
C10	0.12111 (18)	0.34965 (15)	−0.01103 (8)	0.0613 (4)	
N11	0.07881 (19)	0.32285 (16)	−0.07828 (7)	0.0901 (5)	
C12	−0.0236 (2)	0.2319 (2)	−0.07878 (9)	0.0898 (6)	
H12A	−0.0746	0.1943	−0.1200	0.108*	
C13	0.22591 (18)	0.44337 (13)	0.01956 (8)	0.0593 (4)	
C14	0.2713 (2)	0.52658 (16)	−0.02812 (10)	0.0767 (5)	
H14A	0.2363	0.5179	−0.0771	0.092*	
C15	0.3661 (3)	0.62072 (17)	−0.00440 (13)	0.0892 (6)	
H15A	0.3955	0.6748	−0.0371	0.107*	
C16	0.4173 (3)	0.63497 (17)	0.06724 (13)	0.0990 (7)	
H16A	0.4813	0.6992	0.0835	0.119*	
C17	0.3742 (2)	0.55401 (15)	0.11556 (10)	0.0850 (6)	
H17A	0.4091	0.5648	0.1644	0.102*	
C18	0.28031 (18)	0.45720 (13)	0.09299 (8)	0.0593 (4)	
C19	0.24338 (18)	0.37467 (13)	0.14869 (8)	0.0570 (4)	
O20	0.28866 (11)	0.25451 (8)	0.14949 (5)	0.0531 (3)	
C21	0.24141 (16)	0.20247 (13)	0.20772 (7)	0.0522 (4)	
C22	0.1756 (2)	0.29195 (15)	0.23785 (8)	0.0685 (5)	
H22A	0.1339	0.2821	0.2785	0.082*	
N23	0.17751 (17)	0.40188 (12)	0.20054 (7)	0.0730 (4)	
C24	0.27167 (16)	0.07253 (13)	0.22396 (7)	0.0516 (4)	
C25	0.18402 (19)	0.00816 (16)	0.26323 (8)	0.0676 (4)	
H25A	0.1055	0.0477	0.2775	0.081*	
C26	0.2127 (2)	−0.11355 (16)	0.28108 (9)	0.0779 (5)	
H26A	0.1547	−0.1562	0.3079	0.093*	
C27	0.3264 (2)	−0.17102 (13)	0.25913 (9)	0.0783 (5)	
H27X	0.3449	−0.2537	0.2710	0.094*	0.627 (3)
C28	0.4143 (2)	−0.11109 (16)	0.22020 (9)	0.0731 (5)	
H28A	0.4919	−0.1521	0.2059	0.088*	
C29	0.38663 (13)	0.01038 (10)	0.20252 (6)	0.0603 (4)	
H29A	0.4458	0.0518	0.1757	0.072*	
F1	−0.37996 (13)	−0.12266 (10)	0.10536 (6)	0.1162 (10)	0.627 (3)
F1A	0.35448 (13)	−0.28464 (10)	0.27722 (6)	0.1097 (15)	0.373 (3)

### Atomic displacement parameters (Å^2^)

**Table:**

	*U*^11^	*U*^22^	*U*^33^	*U*^12^	*U*^13^	*U*^23^
C2	0.0755 (13)	0.0850 (13)	0.0943 (14)	−0.0163 (10)	0.0260 (11)	−0.0062 (11)
C3	0.0700 (13)	0.1112 (15)	0.0847 (14)	−0.0244 (11)	0.0053 (11)	−0.0203 (11)
C4	0.0627 (11)	0.1125 (15)	0.0551 (9)	−0.0099 (10)	−0.0005 (9)	−0.0114 (9)
C5	0.0493 (9)	0.0709 (9)	0.0482 (8)	0.0065 (7)	0.0046 (7)	−0.0034 (7)
C6	0.0745 (11)	0.0633 (9)	0.0536 (9)	−0.0050 (8)	0.0123 (8)	−0.0077 (7)
C7	0.0945 (14)	0.0753 (11)	0.0638 (10)	−0.0066 (10)	0.0252 (10)	−0.0043 (9)
C8	0.0528 (9)	0.0831 (10)	0.0445 (8)	0.0028 (8)	0.0022 (7)	−0.0033 (7)
O9	0.0592 (6)	0.0682 (6)	0.0411 (5)	0.0030 (5)	0.0076 (5)	0.0026 (4)
C10	0.0634 (10)	0.0778 (10)	0.0439 (8)	0.0072 (8)	0.0129 (7)	0.0120 (7)
N11	0.0927 (12)	0.1320 (14)	0.0450 (8)	−0.0220 (11)	0.0112 (8)	0.0085 (8)
C12	0.0843 (13)	0.1395 (17)	0.0429 (9)	−0.0238 (13)	0.0049 (9)	−0.0017 (10)
C13	0.0646 (10)	0.0579 (9)	0.0569 (9)	0.0104 (7)	0.0154 (8)	0.0087 (7)
C14	0.0890 (13)	0.0739 (11)	0.0701 (11)	0.0085 (10)	0.0221 (10)	0.0202 (9)
C15	0.1089 (16)	0.0629 (11)	0.1017 (16)	0.0036 (11)	0.0347 (13)	0.0229 (10)
C16	0.1239 (19)	0.0595 (11)	0.1153 (18)	−0.0168 (11)	0.0265 (15)	0.0006 (11)
C17	0.1146 (16)	0.0605 (10)	0.0780 (12)	−0.0085 (11)	0.0124 (11)	−0.0034 (9)
C18	0.0709 (11)	0.0502 (8)	0.0574 (9)	0.0084 (8)	0.0136 (8)	0.0009 (7)
C19	0.0665 (10)	0.0543 (9)	0.0491 (8)	0.0066 (7)	0.0074 (7)	−0.0036 (7)
O20	0.0637 (6)	0.0552 (6)	0.0401 (5)	0.0056 (5)	0.0089 (5)	0.0005 (4)
C21	0.0564 (9)	0.0641 (9)	0.0351 (7)	0.0010 (7)	0.0055 (6)	−0.0003 (6)
C22	0.0850 (12)	0.0756 (11)	0.0490 (9)	0.0089 (9)	0.0230 (9)	0.0005 (8)
N23	0.0956 (11)	0.0684 (8)	0.0592 (8)	0.0146 (8)	0.0251 (8)	−0.0016 (7)
C24	0.0571 (9)	0.0620 (8)	0.0327 (6)	−0.0013 (7)	0.0005 (6)	−0.0014 (6)
C25	0.0741 (11)	0.0758 (10)	0.0542 (9)	0.0029 (9)	0.0154 (8)	0.0047 (8)
C26	0.0976 (14)	0.0739 (11)	0.0631 (10)	−0.0092 (11)	0.0175 (10)	0.0124 (9)
C27	0.1052 (15)	0.0623 (10)	0.0629 (11)	0.0043 (10)	0.0037 (11)	0.0084 (8)
C28	0.0789 (12)	0.0714 (11)	0.0673 (11)	0.0136 (9)	0.0088 (9)	−0.0004 (8)
C29	0.0626 (10)	0.0689 (10)	0.0480 (8)	0.0003 (8)	0.0066 (7)	−0.0003 (7)
F1	0.1184 (17)	0.1224 (17)	0.1149 (16)	−0.0571 (13)	0.0399 (13)	−0.0033 (12)
F1A	0.144 (3)	0.0654 (19)	0.121 (3)	0.0135 (18)	0.030 (2)	0.0228 (16)

### Geometric parameters (Å, °)

**Table:**

F1—C2	1.293 (2)	C15—C16	1.362 (3)
F1A—C27	1.290 (2)	C15—H15A	0.9300
C2—C3	1.364 (3)	C16—C17	1.379 (3)
C2—C7	1.368 (2)	C16—H16A	0.9300
C2—H2X	0.9300	C17—C18	1.381 (2)
C3—C4	1.364 (2)	C17—H17A	0.9300
C3—H3A	0.9300	C18—C19	1.470 (2)
C4—C5	1.384 (2)	C19—N23	1.2842 (18)
C4—H4A	0.9300	C19—O20	1.3658 (16)
C5—C6	1.384 (2)	O20—C21	1.3806 (15)
C5—C8	1.442 (2)	C21—C22	1.329 (2)
C6—C7	1.371 (2)	C21—C24	1.4553 (19)
C6—H6A	0.9300	C22—N23	1.3851 (19)
C7—H7A	0.9300	C22—H22A	0.9300
C8—C12	1.342 (2)	C24—C29	1.3869 (18)
C8—O9	1.3755 (18)	C24—C25	1.389 (2)
O9—C10	1.3530 (17)	C25—C26	1.373 (2)
C10—N11	1.296 (2)	C25—H25A	0.9300
C10—C13	1.452 (2)	C26—C27	1.358 (3)
N11—C12	1.370 (2)	C26—H26A	0.9300
C12—H12A	0.9300	C27—C28	1.362 (3)
C13—C14	1.394 (2)	C27—H27X	0.9300
C13—C18	1.399 (2)	C28—C29	1.3688 (19)
C14—C15	1.369 (3)	C28—H28A	0.9300
C14—H14A	0.9300	C29—H29A	0.9300
			
F1—C2—C3	117.53 (17)	C16—C15—H15A	120.1
C28—C27—F1A	118.98 (16)	C14—C15—H15A	120.1
F1—C2—C7	121.08 (18)	C15—C16—C17	119.9 (2)
C26—C27—F1A	119.12 (16)	C15—C16—H16A	120.1
C3—C2—C7	121.38 (15)	C17—C16—H16A	120.1
C3—C2—H2X	119.3	C16—C17—C18	121.41 (18)
C7—C2—H2X	119.3	C16—C17—H17A	119.3
C2—C3—C4	119.32 (17)	C18—C17—H17A	119.3
C2—C3—H3A	120.3	C17—C18—C13	118.84 (15)
C4—C3—H3A	120.3	C17—C18—C19	117.16 (15)
C3—C4—C5	121.03 (16)	C13—C18—C19	123.99 (14)
C3—C4—H4A	119.5	N23—C19—O20	113.56 (12)
C5—C4—H4A	119.5	N23—C19—C18	128.11 (13)
C6—C5—C4	118.31 (15)	O20—C19—C18	118.25 (12)
C6—C5—C8	121.79 (14)	C19—O20—C21	104.70 (10)
C4—C5—C8	119.90 (14)	C22—C21—O20	106.61 (12)
C7—C6—C5	120.87 (15)	C22—C21—C24	134.38 (13)
C7—C6—H6A	119.6	O20—C21—C24	119.01 (11)
C5—C6—H6A	119.6	C21—C22—N23	110.87 (13)
C2—C7—C6	119.08 (16)	C21—C22—H22A	124.6
C2—C7—H7A	120.5	N23—C22—H22A	124.6
C6—C7—H7A	120.5	C19—N23—C22	104.25 (12)
C12—C8—O9	106.02 (15)	C29—C24—C25	118.39 (14)
C12—C8—C5	135.87 (16)	C29—C24—C21	122.43 (12)
O9—C8—C5	118.11 (12)	C25—C24—C21	119.17 (13)
C10—O9—C8	105.62 (11)	C26—C25—C24	120.40 (16)
N11—C10—O9	112.90 (15)	C26—C25—H25A	119.8
N11—C10—C13	127.55 (14)	C24—C25—H25A	119.8
O9—C10—C13	119.52 (13)	C27—C26—C25	119.39 (16)
C10—N11—C12	104.68 (14)	C27—C26—H26A	120.3
C8—C12—N11	110.77 (16)	C25—C26—H26A	120.3
C8—C12—H12A	124.6	C26—C27—C28	121.88 (14)
N11—C12—H12A	124.6	C26—C27—H27X	119.1
C14—C13—C18	118.53 (16)	C28—C27—H27X	119.1
C14—C13—C10	116.98 (14)	C27—C28—C29	118.98 (16)
C18—C13—C10	124.47 (13)	C27—C28—H28A	120.5
C15—C14—C13	121.44 (18)	C29—C28—H28A	120.5
C15—C14—H14A	119.3	C28—C29—C24	120.96 (13)
C13—C14—H14A	119.3	C28—C29—H29A	119.5
C16—C15—C14	119.88 (18)	C24—C29—H29A	119.5
			
F1—C2—C3—C4	179.65 (17)	C16—C17—C18—C13	−1.4 (3)
F1—C2—C7—C6	−179.66 (16)	C16—C17—C18—C19	178.80 (17)
C7—C2—C3—C4	−0.1 (3)	C14—C13—C18—C17	1.3 (2)
C2—C3—C4—C5	−0.7 (3)	C10—C13—C18—C17	−176.83 (16)
C3—C4—C5—C6	1.4 (3)	C14—C13—C18—C19	−178.87 (15)
C3—C4—C5—C8	−179.17 (16)	C10—C13—C18—C19	3.0 (2)
C4—C5—C6—C7	−1.4 (2)	C17—C18—C19—N23	62.1 (2)
C8—C5—C6—C7	179.17 (15)	C13—C18—C19—N23	−117.72 (19)
C3—C2—C7—C6	0.1 (3)	C17—C18—C19—O20	−114.53 (16)
C5—C6—C7—C2	0.7 (3)	C13—C18—C19—O20	65.7 (2)
C6—C5—C8—C12	−178.5 (2)	N23—C19—O20—C21	1.55 (17)
C4—C5—C8—C12	2.1 (3)	C18—C19—O20—C21	178.63 (13)
C6—C5—C8—O9	1.8 (2)	C19—O20—C21—C22	−1.09 (15)
C4—C5—C8—O9	−177.70 (14)	C19—O20—C21—C24	179.70 (13)
C12—C8—O9—C10	−0.55 (17)	O20—C21—C22—N23	0.37 (18)
C5—C8—O9—C10	179.30 (13)	C24—C21—C22—N23	179.41 (16)
C8—O9—C10—N11	0.18 (18)	O20—C19—N23—C22	−1.31 (19)
C8—O9—C10—C13	178.55 (13)	C18—C19—N23—C22	−178.03 (16)
O9—C10—N11—C12	0.3 (2)	C21—C22—N23—C19	0.6 (2)
C13—C10—N11—C12	−177.95 (17)	C22—C21—C24—C29	−154.69 (17)
O9—C8—C12—N11	0.7 (2)	O20—C21—C24—C29	24.3 (2)
C5—C8—C12—N11	−179.07 (18)	C22—C21—C24—C25	24.4 (3)
C10—N11—C12—C8	−0.6 (2)	O20—C21—C24—C25	−156.70 (13)
N11—C10—C13—C14	10.2 (3)	C29—C24—C25—C26	1.0 (2)
O9—C10—C13—C14	−167.91 (14)	C21—C24—C25—C26	−178.13 (15)
N11—C10—C13—C18	−171.60 (17)	C24—C25—C26—C27	−0.9 (3)
O9—C10—C13—C18	10.3 (2)	C25—C26—C27—C28	0.5 (3)
C18—C13—C14—C15	−0.4 (3)	C26—C27—C28—C29	−0.3 (3)
C10—C13—C14—C15	177.86 (16)	C27—C28—C29—C24	0.3 (2)
C13—C14—C15—C16	−0.5 (3)	C25—C24—C29—C28	−0.7 (2)
C14—C15—C16—C17	0.4 (3)	C21—C24—C29—C28	178.37 (13)
C15—C16—C17—C18	0.5 (3)		

### Hydrogen-bond geometry (Å, °)

**Table:**

*D*—H···*A*	*D*—H	H···*A*	*D*···*A*	*D*—H···*A*
C16—H16A···F1^i^	0.93	2.31	3.235 (2)	170
C28—H28A···F1^ii^	0.93	2.45	3.155 (2)	133

Symmetry codes:  (i) *x*+1, *y*+1, *z*; (ii) *x*+1, *y*, *z*.

### Table 2 π–π interactions (Å, °)

*Cg*1, *Cg*2 and *Cg*3 are the centroids of the C2–C7, O9/C8/C12/N11/C10 and C13–C18 rings, respectively. *CgI*··· *CgJ* is the distance between the ring centroids. The interplanar angle is that between the planes of rings *I* and *J*. *CgI*_Perp is the perpendicuar distance of *CgI* from ring *J*. *CgI*_Offset is the distance between *CgI* and the perpendicular projection of *CgJ* on the ring *I*.

**Table:**

*I*	*J*	*CgI*···*CgJ*	Interplanar angle	*CgI*_Perp	Cg*I*_Offset
2	1^iii^	3.818 (1)	2.0 (1)	3.505 (1)	1.514
1	2^iii^	3.818 (1)	2.0 (1)	3.546 (1)	1.415
2	3^iv^	3.860 (1)	10.9 (1)	3.803 (1)	0.661
3	2^iv^	3.860 (1)	10.9 (1)	3.762 (1)	0.864

Symmetry codes: (iii) -x, -y, -z; (iv) -x, -y+1, -z.
